# Supplementary material for: Are carbon emissions trading and green financial instruments synergistic? -Comprehensive quantitative research based on content analysis
Source: PLoS One. 2024 Mar 7;19(3):e0298601. doi: 10.1371/journal.pone.0298601 (PMC10919601; doi:10.1371/journal.pone.0298601)
Supplement: S1 Data — (ZIP) [file pone.0298601.s001.zip › Data/地级市Z_F工作报告.zip!/╣π╢1⁄2/╔ε█┌╩╨/└┤╫╘╣1⁄2╓┌║┼í╛┬φ┐╦╩2╛▌═°í┐_2010.docx]

各位代表：

　　现在，我代表市人民政府向大会作政府工作报告，请予审议，并请各位政协委员和其他列席人员提出意见。

　　一、过去五年工作回顾

　　过去五年，全市人民在党中央国务院、省委省政府和市委的正确领导下，以邓小平理论和“三个代表”重要思想为指导，以科学发展观统领全局，解放思想，改革创新，团结一心，努力工作，推动经济、政治、文化、社会和生态文明建设取得全面进步，圆满完成了市四届人大一次会议确定的各项目标任务。

　　（一）过去五年，综合经济实力稳步增长。

　　五年来，我们采取各种积极有效的措施，努力克服国际金融危机等不利因素影响，推动综合经济实力跨上新台阶。本市生产总值年均增长13.8%，2009年达到8201亿元，居内地大中城市第四位；人均生产总值达到13581美元，提前两年实现“十一五”规划目标。工业增加值年均增长14.3%，达到3598亿元。全社会固定资产投资年均增长9.4%，达到1709亿元。社会消费品零售总额年均增长15.8%，达到2599亿元。全口径财政收入年均增长18.5%，达到2765亿元，其中地方财政一般预算收入达到880.8亿元，比2004年增长173.9%。5年累计实现中央级财政收入8153亿元，服务全国的能力显著增强。在中国社科院发布的城市竞争力评估报告中，深圳综合竞争力连续四年列内地城市榜首。

　　（二）过去五年，发展方式转变初见成效。

　　自主创新实现新突破。国家创新型城市建设全面启动，区域创新体系进一步完善，全社会研发投入占GDP比重达到3.6%。源头创新能力显著增强，中科院深圳先进技术研究院、国家超级计算深圳中心、深圳华大基因研究院，以及数字音频编解码技术、电子信息产品协同互联、电子信息产品标准化国家工程实验室等一批重大科研机构和创新基地落户深圳，深圳清华大学研究院、深港产学研基地等机构创新能力进一步增强。“深港创新圈”建设深入推进，深港科技合作不断加强。专利申请量及授权量快速增长，其中发明专利申请量年均增长37.8%，发明专利授权量年均增长62.5%。2009年PCT国际专利申请量占全国的比重达到47.5%，连续六年居全国首位。

　　产业结构进一步优化。2009年三次产业结构比例为0.1∶46.7∶53.2，第三产业比重比2004年提高5.1个百分点。以华为、中兴、比亚迪、腾讯为代表的高新技术企业发展迅速，华星光电8.5代液晶面板等一批重大产业项目开工或投产，生物、新能源、互联网等战略性新兴产业发展态势良好。高新技术产品产值达到8508亿元，比2004年增长160.5%，其中拥有自主知识产权的比重达到59.5%。金融业快速发展，金融业总资产达到 3.29万亿元，增长197.5%，金融机构本外币存、贷款余额达到1.84万亿元和1.48万亿元，分别增长125.4%和124.1%。创业板正式推出，多层次资本市场体系进一步完善。我市在中小板和创业板上市企业达到37家，占两板上市企业数的11%。创新融资担保方式，中小企业融资环境得到改善。证券、基金、创投业继续保持全国领先地位，全国保险创新发展试验区建设取得积极成果，区域金融中心地位进一步巩固。现代物流业持续发展，深圳港集装箱吞吐量连续七年居全球第四，机场旅客吞吐量连续八年居内地城市第四位。文化产业蓬勃发展，一批龙头文化企业发展态势良好，走出了一条文化与科技、金融、旅游融合发展的新路。深圳被联合国教科文组织评为“设计之都”，“高交会”、“文博会”、“国际人才交流大会”等品牌展会的国际影响力进一步扩大。

　　经济增长的质量和效益明显提高。每平方公里土地产生4.12亿元的生产总值和1.42亿元的财政收入，比2004年分别提高1.93亿元和0.81亿元。循环经济发展取得积极成效，被列为国家循环经济试点城市，单位生产总值能耗、水耗持续下降，在全国保持领先水平。

　　（三）过去五年，改革开放不断突破。

　　改革迈出新步伐。国务院批准《珠江三角洲地区改革发展规划纲要（2008—2020年）》和《深圳市综合配套改革总体方案》，明确深圳“一区四市”的战略定位和四个“先行先试”的改革方向，深圳正式成为国家综合配套改革试验区。国资国企改革深入推进，国有资产质量和效益位居全国前列。完成市区第三轮财政体制改革，市区两级事权与财权进一步匹配。投融资体制改革步伐加快，组建光明、坪山新区城市建设投融资平台，政府投资项目实施并联审批，在地铁建设等领域推行BOT、BT模式。事业单位分类改革基本完成，结束了政府机关和事业单位办企业的历史。不断推动社会管理创新，改革社会组织登记管理体制，建立社会工作制度。设立社区工作站，初步理顺基层管理体制。社区居民自治和服务功能得到强化，居委会直选率从2005年的47%提高到92.8%。积极探索流动人口管理新模式，全面推行居住证制度。

　　对外开放水平不断提高。外贸进出口总额年均增长12.8%，达到2702亿美元，出口实现十七连冠。五年累计实际利用外资181亿美元，来深投资的世界500强企业达到166家。全面实施“走出去”战略，中国—越南（深圳—海防）经贸合作区启动建设，五年来深圳企业累计在境外设立企业和机构405家，协议投资总额27.3亿美元。国际交往进一步扩大，新增国际友好城市2个，友好交流城市16个。深港合作取得重要突破，深港西部通道、福田口岸等一批重大跨界基础设施建成使用。前海深港现代服务业合作区正式启动，创立并成功举办三届深港城市/建筑双城双年展。深莞惠合作全面加强，珠江口东岸一体化进程显著加快，“双转移”工作扎实推进，服务全省的能力不断增强。积极对口帮扶贵州、西藏、新疆及河源、湛江等地区和城市，高质量开展对口支援甘肃地震灾区恢复重建工作，被誉为全国对口支援工作的一面旗帜。

　　（四）过去五年，城市建设快速发展。

　　城市规划力度不断加大。制定实施《深圳2030城市发展策略》，开展新一轮城市总体规划和土地利用总体规划编制，将全市近一半的土地划入基本生态控制线，城市规划的战略性、前瞻性不断增强。农村城市化全面完成，光明、龙华、大运、坪山四大新城建设正式启动，特区内外一体化进程明显加快，城市空间结构不断优化。内伶仃岛归属我市管辖。

　　城市功能不断增强。国家铁路“两线三站”建设加快，机场扩建工程稳步推进，铜鼓航道正式通航，大铲湾港区建成开港，城市辐射力明显增强。地铁1—5号线土建工程基本完成，“一横八纵”城市骨干路网基本形成，城市交通功能提升。水电油气等资源能源工程有序推进，城市保障能力进一步提高。

　　城市面貌不断改善。持续开展“城市管理年”与“基层基础年”、“清无”、“查违”和市容环境提升行动，市容环境明显改善。五年新建垃圾转运站423座，公厕403个，环卫保洁水平进一步提高。城市更新步伐加快，出台城市更新办法，城中村和旧工业区改造稳步推进。落实市人大《关于坚决查处违法建筑和违法用地的决定》、《关于农村城市化历史遗留违法建筑的处理决定》，违法建筑和违法用地普查工作基本完成，查处工作有序推进，历史遗留问题逐步得到解决。

　　生态文明水平不断提升。制定《深圳生态文明建设行动纲领（2008—2010）》，实施生态文明建设系列工程。深入治理水、大气、噪声和固体废弃物污染，化学需氧量、二氧化硫排放量分别削减24.2%和26.9%。生态市建设有序推进，被列为全国首批生态文明建设试点地区和首个国家生态园林示范城市。五年新建公园435个，“公园之城”初步形成。

　　（五）过去五年，社会民生持续改善。

　　人民生活水平进一步提高。人均可支配收入达到2.92万元，比2004年增长47.9%，企业退休人员养老待遇五年提高76.6%。城镇居民登记失业率保持在3%以内，“零就业家庭”户数保持动态归零。社会保障体系不断完善，建立农民工医保、少儿医保、大学生医保制度，在全国率先基本实现“全民医保”。农民工的养老、工伤和医疗保险参保人数均名列内地大中城市之首。初步构建起多层次住房保障体系，推动出台《深圳市保障性住房条例》，“十一五”期间安排建设保障性住房16.7万套，解决1.85万户户籍低收入家庭住房困难，低保户住房保障实现应保尽保，启动实施人才安居工程。

　　教育、卫生、体育事业实现新发展。新增基础教育学位25.4万个，完成特区外96所原村办小学改扩建工程。全面推行免费义务教育，惠及学生176万人次。高等教育发展迅速，南方科技大学筹建取得重大突破，深圳大学、深圳职业技术学院、深圳信息职业技术学院教学科研水平进一步提高，清华大学、北京大学、哈尔滨工业大学深圳研究生院成为我市创建国家创新型城市的重要力量。新型城市医疗服务体系建设全面推进，市第三人民医院、疾控中心等一批重大卫生项目建成使用，社区健康服务网络实现全覆盖，基本公共卫生服务均等化水平大幅提高。卫生应急机制不断完善，有效防控甲型流感疫情。成功申办第26届世界大学生夏季运动会，大运场馆建设等各项筹办工作有序展开，大运会城市行动计划进展顺利。

　　精神文明建设取得新突破。我市连续两次被评为“全国文明城市”，涌现出以丛飞、郭春园为代表的一大批先进人物。图书馆新馆、博物馆新馆、音乐厅、中心书城、24小时街区自助图书馆等一批重点文化设施投入使用，公益性文化场馆在全国率先免费开放，“深圳读书月”、“公园文化节”等品牌文化活动蓬勃开展。

　　其他各项社会事业取得新进步。食品安全“五大工程”、“四大体系”初步建成，药品安全专项整治成效明显，市民饮食用药安全水平进一步提升。高度重视应急管理、安全生产、信访维稳和社会治安工作，社会安全形势总体保持稳定。新气象台、天文台等公共设施建成使用，气象服务水平进一步提高。人口计生、统计、档案、民族、宗教、司法、外事、港澳、台务、侨务、民防、防震减灾等各项事业都取得新进步，社会福利、慈善、老年人、妇女儿童和残疾人事业继续发展，民兵预备役和军民共建工作扎实推进，连续第四次荣获“全国双拥模范城”称号。

　　（六）过去五年，政府建设扎实推进。

　　政府自身建设取得重要突破。积极探索决策、执行、监督相互制约又相互协调的行政运行机制，按照“市场、效率、服务”的导向，实施新一轮政府机构改革，初步建立起职能有机统一的大部门体制，共减少15个政府部门，取消、调整1/3的行政审批事项。实施功能区发展战略，设立光明新区、坪山新区和前海深港现代服务业合作区。积极开展公务员分类管理改革和聘任制公务员试点工作，公安系统公务员专业化改革深入推进。开展第四轮行政审批制度改革，创新行政审批运作方式和运行机制，全面加强政府系统反腐倡廉建设，推进政府绩效管理和行政电子监察，行政效率和执行力进一步提高。

　　民主法治建设继续推进。认真执行法律法规和人大各项决议，主动向市人大、政协报告、通报工作，自觉接受人大的法律监督、工作监督和人民政协、民主党派的民主监督。五年来，市政府系统共办理代表大会议案1件、代表重点建议10件、建议2963件，政协重点提案和建议案22件、提案3128件。大力推进依法行政，在全国率先颁布实施《法治政府建设指标体系》，提请市人大审议法规议案42项，制定、修改和废止了一批政府规章。在全国率先清理非行政许可审批登记事项，非行政许可事项减少50%。加快推进政务公开和电子政务建设，政府工作的透明度不断提高。

　　各位代表！过去五年，我们所取得的成绩和进步，是党中央国务院、省委省政府和市委坚强领导的结果，凝聚了全市人民的辛勤劳动和聪明才智。在此，我代表深圳市人民政府，向奋斗在各条战线的全市广大干部群众及外地来深建设者，向给予我们支持的人大代表、政协委员、各民主党派、工商联和社会各界人士，向中央和各省区市驻深单位，驻深解放军、武警官兵，向所有关心和支持深圳发展的港澳同胞、台湾同胞、海外侨胞和国际友人表示崇高的敬意和衷心的感谢！

　　在看到成绩的同时，我们也清醒地认识到，深圳经济社会发展仍然存在一些深层次矛盾和突出问题。一是资源、环境约束趋紧，发展方式转型压力加大，经济发展质量效益还不够高。二是特区内外发展不协调，二元结构依然突出，城市现代化水平需要进一步提升。三是城市国际化水平不够高，国际知名度、影响力和吸引集聚国际资源的能力有待提升。四是城市人口快速扩张与社会发展相对不足的矛盾比较突出，在住房、交通、教育、医疗、治安等民生领域还存在不少问题，不能很好地满足广大市民群众的期望。五是改革正处于攻坚期，一些重点领域和关键环节改革力度还不够大，改革的科学性、系统配套性亟需增强。六是政府职能转变与作风建设仍与建设民生型、服务型政府存在差距；一些领导干部腐败案件影响政府公信力与形象。以上问题事关长远，影响全局，我们将高度重视，采取切实有效的措施，认真加以解决。

　　二、今后五年目标与任务

　　各位代表，今年是深圳经济特区建立30周年。刚刚召开的中共深圳市第五次党代会确立了深圳发展的重大方略，描绘了深圳未来30年的壮丽蓝图。今后五年是深圳新30年的第一个五年，对深圳未来发展至关重要。深圳经过30年的努力奋斗，在自主创新、产业发展、城市建设和社会民生等多个领域已经打下了很好的基础，创新创业环境优良，具备了把握先机、调整路径、转变方式，再上新台阶、实现新突破的条件和实力。然而，我们十分清醒地认识到，世界经济仍然存在诸多不确定性，国内经济社会发展中一些矛盾和问题更加突出，区域竞争也日趋激烈。面对机遇与挑战，我们必须进一步振奋精神、坚定信心，切实增强紧迫感、危机感和使命感，努力激发全市干部群众干事创业的豪情壮志，抢抓机遇，争先进位，推动科学发展引领全国之先，发展方式转变引领全国之先，再创特区新的辉煌。

　　未来五年政府工作的指导思想是：高举中国特色社会主义伟大旗帜，以邓小平理论和“三个代表”重要思想为指导，深入贯彻落实科学发展观，按照《珠江三角洲地区改革发展规划纲要（2008—2020年）》的要求和市第五次党代会的统一部署，以创新发展、转型发展、低碳发展、和谐发展为导向，追求“好”、力争“快”、坚持“特”、突出“新”、立足“干”，率先转变发展方式，全面优化发展环境，深入推进改革开放，着力改善社会民生，推动经济、政治、文化、社会和生态文明建设全面协调发展，努力当好科学发展排头兵，加快建设现代化国际化先进城市。

　　未来五年政府工作的基本思路是：立足新起点、瞄准新目标、走出新路子、实现新发展。

　　——立足新起点。深圳经济特区已经进入“三十而立”之年，综合实力跻身内地大城市前列，经济社会发展正处于加快转型的关键时期。中央和省确立了深圳“一区四市”的战略定位，赋予了深圳当好科学发展排头兵的新的历史使命，深圳正站在新的历史起点上。

　　——瞄准新目标。敢为天下先是深圳的风格，追求卓越是深圳的境界。我们将放宽视野，学习借鉴国内先进城市经验，不断提高城市现代化水平；我们将提升眼界，以香港、新加坡等世界先进城市为标杆，不断提高城市国际化水平和国际影响力。

　　——走出新路子。我们将加快转变发展方式，推动经济增长从外需拉动向内外需协调拉动转变，从要素驱动向创新驱动转变，从注重经济发展向推动经济、政治、文化、社会、生态协调发展转变，从偏重物质进步向更加关注人的全面发展转变，走出一条科学发展的新路子。

　　——实现新发展。我们要解放思想，改革创新，锐意进取，力争在短期内使深圳焕发新精神、展示新面貌、再创新优势、实现新发展，努力建设国际竞争力更强、创新活力更足、发展环境更优、民生福利更高的现代化国际化先进城市。到2015年，本市生产总值超过1.5万亿元，接近新加坡的总量水平；人均生产总值超过2万美元，达到中等发达国家水平；居民人均可支配收入达到4.9万元。

　　未来五年政府工作将主要围绕四个方面展开：

　　（一）率先转变发展方式，走出科学发展的新路子。

　　要在科学发展的道路上领跑，深圳必须打破传统发展方式的束缚，加快经济结构调整，尊重资源环境的约束，谋发展、做决策必须综合考虑资源环境承载力，着力提高资源利用效率，力求以更小的投入、更低的消耗，创造更大的效益，真正走出一条科学发展的新路，率先进入更高的发展境界。

　　一是大力推进自主创新，增强经济增长的主动力。积极建设国家创新型城市，将自主创新作为转变发展方式的中心环节和主导战略，把核心技术创新作为自主创新的主攻方向，积极推进原始创新、引进消化吸收再创新和集成创新。加快实施重大科技专项和重点技术攻关，力争在通信、新能源、基因技术等领域达到世界领先水平，把深圳打造成核心技术创新之城。积极引进国内外先进的科研教育机构、国家重点实验室、工程实验室、技术检测实验室，加强公共技术服务平台建设。深入推进“深港创新圈”布局发展，完善区域创新体系，促进区域创新资源聚集整合，实现开放式创新及创新升级。鼓励发展创业投资、风险投资，进一步完善技术产权交易市场。大力实施知识产权战略和标准化战略，在一些重点行业和优势产业主导制定一批国际标准，抢占国际经济和科技竞争的制高点。

　　二是加快产业结构调整，提升经济国际竞争力。把握经济全球化时代产业链、价值链跨区域、跨领域融合发展的趋势，大力调整产业结构，着力构建“高端化、总部型、低碳化、集群化”的现代产业体系。做强做精第二产业，巩固制造业在城市经济中的支撑地位；加快发展第三产业，提升第三产业发展水平，实现二、三产业良性互动。坚持高端化发展方向，大力发展高新技术产业，推动企业向价值链的研发设计、品牌服务等高端环节攀升。努力营造良好的金融生态环境，发展多层次资本市场，充分利用创业板等各种有利因素推动企业上市融资。加快金融改革创新综合试验区、保险创新发展试验区建设，完善金融配套服务，扩大跨境贸易人民币结算业务，巩固和提升深圳区域金融中心的地位，进一步提高金融对经济的服务能力和贡献率。继续做大做强文化、物流、旅游、会展等高端服务业，积极发展海洋产业和都市农业。大力发展总部经济，吸引国内外企业集团总部和区域总部、研发中心、采购中心、营运中心落户深圳。坚持低碳化发展方向，大力开发低碳技术，加快发展新能源和可再生能源，促进能源资源集约节约利用。坚持集群化发展方向，以国际一流标准加快高新技术园区和产业集聚基地建设，把深圳打造成世界先进制造业基地。以科技、文化、品牌、电子商务改造提升优势传统产业，推动传统产业优化升级，创造更大的附加价值。促进加工贸易企业就地转型，实现品牌化、集约化发展。加大中小企业扶持力度，优化企业发展环境，积极推动企业改制上市，形成强大的中小企业梯队，促进产业聚变、裂变。

　　三是加快功能区开发建设和新兴产业发展，打造新的经济发展极。深圳经济已经处于一个较高的平台之上，未来要在做大做强存量的同时，积极打造新的区域发展极和新的产业增长点，以增量求发展、求突破。要努力把握产业发展和城市建设规律，科学规划区域布局，合理确定各功能区定位，促进城市统筹协调发展。借鉴先进城市以大项目大投入推动开发建设的经验，创新开发模式，按照“产业集群化、用地集约化”思路，高起点规划、高水平建设光明、坪山、前海等功能区，将其打造成高新技术产业、先进制造业和现代服务业集聚地，成为经济发展的主战场。积极落实各项战略性新兴产业振兴规划和政策，大力发展互联网、新能源、生物三大战略性新兴产业，力争到2015年产值规模超过6500亿元，成为经济发展的生力军。

　　四是继续保持经济较快发展，不断巩固深圳经济中心地位。在推进发展方式转变的同时，要力争保持一定的增长速度，做大经济总量。要坚持内外需并重，积极扩投资、促消费、稳出口，保持经济增长的持续性。以完善城市功能、促进产业发展、改善社会民生、推动特区内外一体化为重点，不断提高固定资产投资的强度和密度。积极推动投融资体制改革，完善城市建设投融资平台，努力拓宽社会投资的领域和范围，激发全社会的投资潜力，力争到2015年累计固定资产投资额超过1.2万亿元。积极扩大居民消费，努力营造安全放心的消费环境，培育旅游、文化、体育、健康等消费热点，完善商业网点布局，创新商业业态，积极打造国际消费中心城市。抓住国家实施扩大内需战略的机遇，充分利用深圳产品的品牌、创新、服务优势，加强内销渠道建设和市场推广力度，以服务内地创造新的发展空间。继续稳定和拓展外需，实施市场多元化战略，巩固传统出口市场，大力开拓东南亚等新兴市场。加快发展服务贸易和服务外包，推动外贸增长方式转变。

　　（二）全面优化发展环境，为科学发展提供强大支撑。

　　从营造发展新优势的高度，以国际先进城市为标杆，以全球眼光和战略思维科学谋划城市发展，不断增强城市的吸引力、竞争力和创造力，为科学发展提供强大支撑。

　　一是坚持科学规划，优化空间环境。提高规划的科学性，充分发挥规划在城市发展中的引导和控制作用，全面优化城市布局结构，为城市发展打基础、拓空间、优环境、增后劲。加快国民经济和社会发展规划、城市总体规划、土地利用总体规划“三规统一”步伐，着力增强各层次、各领域规划的协调性、可实施性，实现法定图则全覆盖。大手笔推进土地资源整合，围绕重点发展地区、产业园区以及重大投资项目，加大土地权属整合力度，将分散的园区和地块进一步整合，提升功能区水平，使其成为吸纳和布局新兴高端产业和项目的重要载体。完善并强化查违工作机制，用五年时间基本完成历史遗留违法建筑和土地问题的处理。创新用地管理机制，加强土地整备储备，严格控制建设用地增量，努力提高土地集约节约利用水平。科学规划和开发城市地下空间、空域和海洋空间，提升城市长远发展的容量。

　　充分利用深圳经济特区扩大到全市的重大机遇，加快推进特区一体化进程，按照国际一流水准，打造区域发展中心。到2015年，光明、龙华、大运、坪山四大新城要初具规模，宝安、龙岗中心区基本成熟，前海、大鹏半岛等地区规划建设迈出实质性步伐。加大特区外基础设施投入和建设力度，提高市政管理和公共服务水平，切实做好城市化过渡期后的体制转轨工作，大幅缩小特区内外发展差距，提高城市整体发展水平。

　　二是夯实城市基础，优化功能环境。加快推进枢纽型、功能性、信息化重大基础设施建设，为建设现代化国际化先进城市提供有力的支撑。加快交通基础设施建设，全面建成广深港客运专线和厦深铁路、穗莞深城际轨道、地铁二期工程、“七横十三纵”干线路网、大铲湾港区、机场二跑道和新航站楼，以及一批重大交通综合枢纽，启动地铁三期工程，加快推进深中通道规划建设，尽快形成覆盖全市主要交通走廊的大容量骨干交通网络，把深圳建成具有全球资源配置功能和国际商务运营功能的全球性物流枢纽城市、亚太地区重要的多式联运中心和供应链管理中心。加强口岸软硬件设施建设，改善口岸通关环境。加快推进城市宽带信息基础设施建设，进一步完善公共信息服务体系，加大信息资源整合利用力度，积极推动电子政务发展，努力打造数字城市、智慧深圳和国际信息枢纽港。

　　不断提高资源能源保障水平。继续推进水源网络建设，研究开辟新的境外水源，加强非传统水资源开发利用，严格节水管理，构筑完善的水资源保障体系。落实电网建设规划，加快重大电力和智能电网工程项目建设，逐步完善电力保障机制。推进重大油气项目建设，拓展油气资源供应渠道，完善油气供应网络和市场应急预警机制。加强粮食等重要物资的储备和调控管理。

　　三是加快更新改造，优化人居环境。坚持新区开发和旧区更新并重，完善城市更新配套政策法规，充分发挥政府和市场的作用，调动全社会的积极性，大力推进城中村和旧住宅区改造，加快旧工业区转型升级，高水平实施旧商业区更新，向城市更新要空间、要资源、要环境。积极开展宜居城市创建工作，深入推进市容环境提升行动，持续提高城市、街景、建筑物的美化、绿化水平，使城市面貌焕然一新。以完善市政配套和环境治理为重点，大力推进城中村综合整治，努力改变城中村“脏、乱、差、挤、危”的落后面貌，逐步提升文明水平。提高重点区域、重点地段和标志性建筑的设计水平，集中打造一批城市亮点和建筑精品，不断提升城市形象和气质。加强文物保护，加大对大鹏所城、客家民居和南头古城等历史街区和传统建筑的保护力度。进一步完善城市管理综合执法体制，创新城市管理手段，强化网格化管理，全面提升城市管理的精细化、规范化和信息化水平。

　　四是加强治理保护，优化生态环境。牢固树立生态建设与保护优先的理念，积极贯彻落实生态文明建设行动纲领，制定实施人居环境工作纲要。加快国家生态文明试点城市和国家低碳生态示范市建设，持续开展生态区、生态工业园区、绿色社区等系列创建活动，推进公共机构节能减排，倡导低碳生活方式，提升全社会生态文明意识，力争成为全国首批“国家生态市”。努力打造区域生态安全体系，扎实推进“四带六廊”生态网络体系和生态湿地系统建设，加快坪山生态湿地园、海上田园生态湿地建设。积极推进绿道网建设，到2012年建成300公里的珠三角区域绿道，2015年形成包括区域绿道、城市绿道和社区绿道的绿道网络。在加强保护的前提下，科学规划、合理利用海洋资源，启动大鹏半岛的生态旅游开发，加强内伶仃岛自然生态保护，加快建设国际滨海城市。建立完善生态补偿机制，加大基本生态控制线监测和保护力度。加强水土保持和危险边坡整治，加快裸露土地复绿，全面修复生态环境。实行最严格的环境保护制度，深入治理水、大气、噪声和固体废弃物污染，推动污水处理厂和污水管网建设取得突破性进展，深入开展蓝天行动，全面推广使用国IV油，到2015年，灰霾天数要明显减少，主要河流水质达到地表水V类标准。深化区域环保合作，建立健全区域河流和大气污染的预警和联防联治机制。

　　五是完善政策体系，优化人才环境。坚持引进、培育与使用并重，大胆创新人才政策法规，创造最优的人才创新创业环境，打造人才“宜聚”城市。努力营造“尊重劳动、尊重知识、尊重创造、尊重人才”的浓厚氛围。做大做强人才载体，尽快形成以企业、科研院所、高校、公共研发和技术服务平台为主体的多层次、多元化的人才集聚平台。制定实施人才发展规划和重大人才工程，以引进培养高层次和高技能专业人才为重点，打造一支门类齐全、规模宏大、梯次合理的高素质人才队伍。

　　六是强化规则意识，优化法治环境。市场经济是法治经济，公民社会更是法治社会。要始终坚持依法治市，强化规则意识，以规则、制度来调整、规范市场经济主体和社会群体的行为及其相互之间的关系，建立良好的市场经济秩序和社会秩序，维护和保障经济社会的正常运行。充分利用特区立法权优势，推动建立和完善与国际通行规则接轨的法规政策体系，努力营造国际化的营商环境。大力弘扬法治精神，深入开展法制宣传教育，在全社会形成尊重法律、尊重规则的良好氛围。加大执法力度，强化执法责任，努力做到规范、公正、文明执法，切实维护法律的权威和尊严。加强诚信制度建设，加快完善企业和个人征信系统，真正形成鼓励诚信、惩戒失信的良好环境。

　　（三）深入推进改革开放，为科学发展注入新的动力。

　　改革开放是深圳的灵魂和生命线，是深圳创造新优势的根本手段。要按照四个“先行先试”的要求，深入落实综合配套改革总体方案，解放思想、勇于创新，不自满、不松懈、不停步。

　　一是全面深化经济体制改革，完善社会主义市场经济体制。坚持社会主义市场经济改革方向，充分发挥市场在资源配置中的基础性作用，加快形成统一开放、竞争有序的市场体系。按照实现基本公共服务均等化的要求，深化市区财政体制改革，加快预算管理制度改革，进一步完善公共财政体系。继续推进国资监管体制和国有企业改革，充分发挥国有经济基础性、先导性和公共性的独特作用，加大产业发展和资本运作力度，推动国有企业做强做大。积极稳妥推进社区股份合作公司改革，促进其规范健康发展。大力扶持民营经济，合理引导民营企业进入金融服务、公用事业、基础设施建设等领域，形成各种所有制经济公平竞争、共同发展的新格局。

　　二是加快行政管理体制改革，提高公共管理水平。加快转变政府职能，实现政府职能向创造良好发展环境、提供优质公共服务、维护社会公平正义转变。创新行政管理体制，深化大部门制改革，形成决策、执行、监督既相互制约又相互协调的行政架构。优化行政运行机制，推动政府管理更加规范有序、公开透明、务实高效。合理划分各层级事权，适当调整行政区划，推进精简行政层级改革试点，缩短管理链条。继续深化行政审批制度改革，大力推进网上审批。全面完成公务员分类管理和聘任制改革，建设一支专业、高效、廉洁的公务员队伍。

　　三是推动社会管理体制改革，不断强化社会管理。创新社会管理体制，整合社会管理资源，健全党委领导、政府负责、社会协同、公共参与的社会管理格局。进一步深化事业单位改革，推进政事分开、管办分开，创新事业单位管理制度和运行机制，加快法定机构建设。推进基层社会管理体制改革，完善社区管理和服务体制，增强基层自治功能。深化社会组织管理体制改革，引导社会组织健康规范发展，增强社会组织参与社会管理和公共服务的功能。不断完善现代社会工作制度，加快培育职业化、专业化、规模化的社工人才队伍。创新人口管理模式，加快建立全口径人口管理和服务机制，加强流动人口管理，合理控制人口规模，不断提高户籍人口比重，优化人口结构。

　　四是实施全方位开放战略，加强国际经济交流与合作。注重“引进来”与“走出去”的有效结合，充分利用国际国内两个市场、两种资源，拓展对外开放的广度和深度。加大招商引资力度，完善机制、整合资源，引进一批重大产业项目，提高利用外资的质量水平。大力实施“走出去”战略，加快中国—越南（深圳—海防）等境外经贸合作园区建设，支持有条件的企业到境外投资发展。加快培育一批跨国公司和国际知名品牌，增强深圳企业参与国际竞争的能力。

　　五是促进区域协调发展，强化深圳中心城市地位。以深港合作为先导、珠江口东岸地区一体化为重点，大力推进区域协调发展，不断扩大深圳经济腹地和国际影响力。全面深化深港、深澳合作，不断创新合作方式、提升合作层次，加快深港重大跨界基础设施建设和前海、河套等地区开发，推动两地产业融合发展，打造优质生活圈，建设与香港共同发展的国际性城市。积极推进珠江口东岸一体化进程，将珠江口东岸地区打造成为珠三角地区改革发展的先行区和示范区。抓住中国-东盟自由贸易区启动的机遇，把深圳打造成为中国与东盟经贸合作的重要节点。不断创新对口支援工作方式，做好对口支援新疆及其它地区的工作。

　　（四）着力改善社会民生，使科学发展成果惠及全市人民。

　　改善民生是经济发展的根本目的。要加快解决市民群众普遍关注、反映强烈的热点难点问题，大力提升民生福利水平，维护社会公平正义，促进社会和谐进步。

　　一是着力提高居民收入和社会保障水平。实施积极的就业政策，完善以创业带动就业的政策体系和工作机制，努力创建充分就业城市和国家级创业型城市，将居民登记失业率控制在3%以内。完善职业培训体系，大力开展公益性技能培训，提升劳动者就业能力。积极构建和谐劳动关系，全面推行劳动合同和集体合同制度，切实维护劳动者的合法权益。完善收入分配制度，建立健全工资正常增长机制，提高劳动报酬在初次分配中的比重。按特区内外统一标准，提高企业最低工资水平。加大收入分配调节力度，不断提高中低收入家庭收入水平，努力缩小不同社会群体的收入差距。

　　按照“广覆盖、保基本、多层次、可持续”的方针，加快完善社会保障体系。健全养老保险制度，做实基本养老保险个人账户，做好养老保险关系跨地区无障碍转移。进一步完善医疗保险、工伤保险和失业保险的服务体系，提高服务水平。规范社保基金管理，确保社保基金保值增值。完善以最低生活保障制度为基础的综合性社会救助体系，大力发展社会福利、慈善、老年人、妇女儿童和残疾人事业。到2015年，力争在全国率先实现“全民社保”。

　　着力提高住房保障水平，认真落实保障性住房条例和人才安居工程政策，在“十一五”期间基本解决户籍低收入家庭住房困难的基础上，“十二五”期间将住房保障重点拓展到中等收入家庭和人才群体。加大对保障性、政策性住房的土地供应、资金投入和建设力度，并充分运用市场机制提高建设效率，着力增加在建项目的开工量、竣工量和供应量。建立健全住房公积金制度，完善住房分配货币化改革，切实提高普通市民的住房支付能力。实施人才安居工程，通过提供购房补贴、租房补贴、公共租赁住房和安居型商品房等方式，解决人才安居问题，全面缓解人才住房困难，优化创新创业环境。贯彻落实物业管理条例，提高物业管理水平，加强物业专项维修资金归集收取，在老住宅区、城中村引入专业化、市场化的物业管理模式，营造安全舒适文明和谐的生活环境。全面加强房地产市场监管，合理引导住房消费，抑制投机行为，促进房地产业健康发展。

　　二是着力发展社会事业。大力实施科教兴市战略，优先发展教育，办好人民满意的教育。探索创新学前教育管理模式，积极发展学前教育。巩固提高义务教育水平，推进义务教育均衡发展，逐步实现常住人口子女平等接受义务教育。加快高中阶段教育发展，不断扩大办学规模，基本解决高中学位不足问题。推动高等教育跨越式发展，全面提高高等教育质量，力争南方科技大学建设初具规模，深圳大学改革发展取得突破，清华大学、北京大学、哈尔滨工业大学深圳研究生院成为国际化、创新性、研究型的高水平大学群。大力发展职业教育，将深圳职业技术学院、深圳信息职业技术学院打造成为全国一流的高等职业院校。加强教育国际交流与合作，加快建设国际学校和港人台商子弟学校，推动与境外高校合作办学取得实质性进展。鼓励社会力量出资办学，推动民办教育规范发展。加快发展继续教育，构建灵活开放的终身教育体系，努力建设人人皆学、处处可学、时时能学的学习型城市。

　　加快推进重大卫生基础设施建设，有效增加基本医疗服务供给。建成滨海医院及其它市级医院，力争全市每千人病床数达到3.9张。深入推进医疗卫生体制改革，完善医疗卫生机构管理体制和运行机制，积极促进非公医疗卫生机构发展，鼓励社会资本参与医疗卫生服务，逐步形成由政府提供公益性基本医疗服务、市场提供个性化和高端医疗服务的格局。完善社区健康服务体系，建立合理就医模式，鼓励社区首诊，提高公共卫生资源使用效率。加强重大疾病防控、医疗救治和卫生监督体系建设，完善职业病防控体系。积极普及心理健康知识，提高市民心理健康水平。大力发展中医事业，推进中医临床研究和中医管理体制改革。

　　加强精神文明建设，继续实施文化立市战略，扎实推进社会主义核心价值体系建设，积极探索中国特色社会主义文明模式，建设市民共有的精神家园。发展公益性文化事业，规划建设一批公共文化设施，完善公共文化服务体系，丰富群众文化生活，提升市民的城市认同感和归属感。打造城市文化品牌，大力推进“两城一都一基地”建设，积极培育龙头文化企业，鼓励文化创新，努力建设文化产业中心城市和先锋城市，提升城市软实力。积极发展体育事业和体育产业，广泛开展全民健身运动。按照“办赛事、办城市，新大运、新深圳”的要求，高水平举办一届更精彩、更成功、更具影响力的世界大学生运动会。扎实推进人口计生、统计、双拥、司法、外事、港澳、台务、侨务、民族、宗教、档案等各项事业。

　　三是着力打造平安深圳。全面强化社会治安综合治理，加快构建全方位、立体化的动态治安防控体系，重点打击各类严重刑事犯罪，保持对“黑恶势力”和“黄赌毒”的严打高压态势。建立完善群防群治体系，加大对社会治安重点地区的集中整治，推动社会治安实现明显好转。加强安全生产工作，扎实做好重点行业和领域的安全监管与隐患排查整治工作，推动落实企业安全生产主体责任，深入开展职业安全教育，杜绝重特大事故发生，确保群众的生命财产安全。加强食品药品安全管理，加大“三防”、气象、民防、防震减灾和地质灾害治理工作力度，提高预防和处置各类突发事件的能力。构建科学有效利益协调机制、诉求表达机制和矛盾调处机制，着力抓好信访维稳工作，积极预防和妥善处置群体性事件，维护群众利益和社会稳定。

　　各位代表！我们将认真接受市人大及其常委会的法律监督和工作监督，坚持依法向市人大常委会报告工作。认真接受市政协的民主监督，加强与市政协的工作沟通和协商，主动听取各民主党派、人民团体和社会各界的意见和建议。切实执行市人大及其常委会通过的各项决议，认真办理人大建议和政协提案、建议案，提高办理工作的质量和实际效果。

　　各位代表！我们将加强政府自身建设，着力打造法治政府、阳光政府、高效政府、廉洁政府、民生政府和服务型政府。我们将按照法治政府指标体系的要求，加快法治政府建设进程，推进政府机构职责、行为、程序的法定化。我们将深入推进政务公开，充分发挥舆论和媒体的监督作用，让权力在阳光下运行。建立健全行政决策机制，完善重大事项集体决策、专家咨询、社会公示听证和决策效果评估制度，提高科学民主决策水平。我们将全面推行政府绩效管理，强化行政问责，优化政务流程，进一步提高行政执行力和行政效能。我们将切实加强廉政建设和反腐败工作，充分发挥监察部门的作用，全面加强审计监督，以制度建设和权力监控为核心，建立健全惩治和预防腐败体系，加大从源头上防治腐败工作力度，坚决查处腐败案件，保持惩治腐败的高压态势，以反腐倡廉的实际成效取信于民，维护政府的良好形象。我们将始终坚持执政为民的理念，把改善民生作为政绩考核的重要标准，把民生福利的指标摆上重要的议事日程，力求在民生服务方面走在全国前列。我们将积极适应新技术、新产业、新商业模式的发展要求和建设国际化城市的需要，不断创新和拓展政府服务的内容和方式，全力提升服务型政府的发展水平，使政府服务成为深圳的新特色、新优势。

　　三、2010年政府主要工作

　　2010年是深圳经济特区建立30周年，也是全面完成“十一五”任务、为“十二五”发展打好基础的关键一年。按照市第五次党代会的总体要求，今年我市经济社会发展的主要预期目标是：本市生产总值增长11%左右；地方财政一般预算收入增长10.1%左右；居民消费价格指数103%左右；城镇登记失业率控制在3%以内；万元GDP能耗、水耗分别下降2.6%和2%；二氧化硫排放总量与上年持平，化学需氧量排放总量下降8%。

　　今年已经过去五个月了，完成全年预期目标任务艰巨，时间紧迫，我们必须鼓足干劲、争分夺秒，重点抓好七个方面的工作：

　　（一）加快转变发展方式。把发展方式转变摆在突出位置，真正在“加快”上下功夫、在“转变”上动真格、在“发展”上见实效。一是推进核心技术创新，增强自主创新能力。加大对基础研究和应用研究的支持力度，组织实施重大科技专项和重点技术攻关。积极推进国家超级计算深圳中心、中科院深圳先进技术研究院等创新平台，以及电子信息产品标准化等国家工程实验室建设。加快组建深圳国家高技术产业创新中心、新一代宽带移动通讯和数字电视国家工程实验室。全面落实《深港创新圈三年行动计划（2009—2011）》，加快深港传染病研究中心等8个创新基地和深港IC设计创新平台等12个服务平台建设。二是加快调整产业结构，提高经济发展质量。巩固制造业在经济中的支撑地位，积极发展金融、物流、会展、文化创意设计等现代服务业。制定出台新材料产业发展规划，加快发展互联网、新能源、生物等战略性新兴产业，抢占产业发展的制高点。加大招商引资力度，积极引进国内外大型企业集团总部、区域总部、研发中心等落户深圳。三是切实推进节能减排，确保完成“十一五”节能减排目标。重点抓好工业、交通、建筑等领域的节能，支持节能重点工程，推动公共机构节能减排。积极发展循环经济和节能环保产业，努力构建以低碳排放为特征的产业体系和消费模式。

　　（二）促进经济平稳较快增长。今年国内外经济形势非常复杂，对深圳经济发展带来很大挑战，我们要积极应对形势变化，采取有效措施，确保完成全年经济增长目标。一是加大对企业的服务支持力度。扎实开展下基层送服务促转变活动，推动政策落实，帮助企业解决用地、融资、人才引进等方面的困难，激发企业经营活力，进一步做大经济存量。二是充分发挥投资的拉动作用。加大对重大基础设施和重大产业项目投资力度，加快推进中芯国际集成电路芯片、清洁能源汽车等重大产业项目建设，以大项目大投入支撑经济大发展。加快投融资体制改革，推动投融资平台建设。落实促进社会投资的政策措施，努力释放全社会的投资活力。三是积极促进居民消费。大力推进福田华强北、罗湖“金三角”等商圈建设，努力扩大大宗商品消费，培育旅游、文化、休闲、健康等新的消费热点，发展信贷消费、网络购物等新型消费业态，推动消费结构优化升级。四是全力稳定出口形势。进一步优化出口产品结构，扩大高新技术产品出口，推动加工贸易转型升级，大力发展服务贸易和服务外包。研究跟踪国际贸易保护新手段，组织指导企业积极应对各类贸易摩擦。实施市场多元化战略，在巩固传统市场的基础上，积极开拓新兴市场。五是大力实施内贸战略。年内举办和组织参加21个国内经贸洽谈会，为深圳企业开拓国内市场搭建平台。全面落实与各省市的战略合作协议，充分利用对口支援甘肃、新疆、贵州等地的机会，拓展经济发展空间。六是加强经济运行分析、监测和预警，提高资源能源保障能力，确保电力供应，做好粮食等大宗商品储备工作。

　　（三）全力解决民生热点难点问题。要把市民群众通过媒体、网络等渠道强烈反映的问题作为改善民生的重点，采取切实举措，认真加以解决。一是提高居民就业和社会保障水平。实施积极的就业政策，重点做好高校毕业生、就业困难人员就业工作，全力创建充分就业城市和国家级创业型城市。进一步提高社会保障水平，推动跨地区就业参保人养老保险关系顺利接续，健全少儿和大学生医保制度。二是要把解决住房难特别是人才住房问题作为住房工作的重点。加快出台《深圳市保障性住房条例》的相关配套实施细则，年内开工建设保障性住房5万套，提供住房1万套，发放5000户住房货币补贴。实施人才安居工程，全面落实人才住房货币化补贴政策，加快启动安居商品房建设。深入落实国家房地产市场调控政策，出台《深圳市房地产市场监管办法》，整顿市场秩序，坚决遏制房价过快上涨，促进房地产市场健康发展。三是加快公共交通网络建设，加大对特区外公交线网开设和运力投放力度，年内开通和调整公交线路100条以上，确保特区外公交覆盖率达80%以上，梅林关、布吉关等交通拥堵问题初步缓解。有序推进轨道交通、北环大道改造等工程，尽量减少城市建设对市民的交通干扰。加大出租车行业整顿力度，加大交通违法行为查处力度，提高公交管理运营水平。四是争创国家教育综合改革示范区，推动南方科技大学建设取得实质性进展，力争今年开始自主招生。加快实施中小学校舍安全工程和改扩建工程，推进寄宿制高中建设，年内新增高中学位9000个。五是大力发展医疗卫生事业，加快医疗卫生体制改革，推进管办分离、医药分开，进一步整顿规范医疗卫生市场秩序，努力解决市民“看病难、看病贵”的问题。六是尽快出台城市更新办法的实施细则，加快推进岗厦、大冲等旧村改造和华强北等城市更新项目。市容环境提升行动实现全面突破，推进街景美化、建筑物与公共设施外立面翻新改造、户外广告整治，加大大气污染治理力度，努力使市容环境质量和城市面貌有较大改观。七是高度重视社会治安问题，加大社会治安综合治理力度，切实加强校园等敏感场所的安全防范工作，防止发生极端个人行为和暴力事件，提高市民群众的安全感。八是更加关心新生代劳务工。努力为一线劳务工尤其是新生代劳务工创造更好的工作、生活和身心发展条件。要积极适应工业化、城市化、现代化快速推进和社会的快速转型，推动企业切实履行社会责任，规范企业劳动用工行为，依法保障员工合法权益，加快构建和谐劳资关系。引导企业树立以人为本的理念，不断改善管理方式，建立健康向上、关爱和谐的企业文化，促进员工人际交流，增强员工集体归属感，使广大劳务工感受到更多的企业情感关怀。充分发挥工会、共青团、妇联等社会组织的引导带动作用，加强对新生代劳务工进行知识、技能和心理健康培训，引导他们热爱劳动、热爱生活、热爱生命，塑造努力工作、不畏挫折、奋发有为的阳光心态。加快政府职能转变，努力为企业和社会特别是“企业型社会”提供更具针对性的服务。进一步提高政府处理企业群体危机事件的应急能力，营造更优的企业发展环境。

　　（四）大力推进重大基础设施建设。推进重大基础设施建设，不仅能为未来发展打基础、做铺垫，更是今年扩大投资，促进经济平稳发展的重要内容。一是加快重大交通基础设施建设，确保年内完成50公里地铁线路试运行。加紧开展轨道交通远景线网规划和建设前期工作，全面启动地铁7、9、11号线建设。加快机场二跑道、飞行区等机场扩建工程建设，有序推进广深港客运专线、厦深铁路、穗莞深城际线、沿江高速、南坪快速二期、丹平路一期、外环高速等重大工程建设。二是推进功能区和重大产业项目建设。加大光明、坪山等功能区土地整备力度，加快华为科技城及各类产业集聚基地建设步伐，推动华星光电、岭澳核电、平安国际金融中心等一批重大产业项目建设。三是大力推进中心公园、当代艺术馆、街区图书馆、数字电视普及、绿道网、城市规划馆等重点民生基础设施建设。四是落实电网建设和改造计划，继续推进供水、国家成品油储备深圳油库、西气东输二线工程等资源能源重大项目建设。

　　（五）不断深化改革开放。认真落实《珠江三角洲地区改革发展规划纲要（2008—2020年）》和《深圳市综合配套改革总体方案》，加快推进重点领域和关键环节的改革。一是加快行政管理体制改革。认真总结推广市政府机构改革的经验做法，积极稳妥地推进区级政府机构改革。探索行政层级改革，在盐田区和光明、坪山新区进行整合街道办及社区工作站试点。推进公务员分类改革试点，扩大聘任制公务员试点范围。全面试行政府绩效管理。完成市区第四轮财政体制改革，充分调动市区两级的积极性。二是全面加强深港合作。加快推进前海深港现代服务业合作区建设和落马洲河套地区开发，加强莲塘/香园围口岸、机场联络线等重大项目建设。进一步深化深港金融合作，积极推动深港跨境贸易人民币结算试点，不断提升深圳区域金融中心地位。三是积极推进珠江口东岸一体化，重点加强与东莞、惠州在发展规划、产业发展、交通运输、环境生态等方面的衔接合作，尽快打通深莞、深惠之间的断头路。扎实推进“双转移”和对口扶贫工作。

　　（六）高质量完成“十二五”规划编制工作。“十二五”时期是我市推动科学发展的机遇期、加快经济转型的关键期和深化改革开放的攻坚期，编制好“十二五”规划至关重要。一是紧紧围绕“一区四市”战略定位，加强重大战略课题研究，科学制定深圳“十二五”总体规划。二是以“统筹兼顾、创新驱动、绿色增长、共建共享”为原则，突出规划重点，深化经济、社会、城市等领域重大专项问题研究，编制新一轮产业、交通、资源、能源、环保、住房、教育、科技、文化等专项规划。三是加强衔接协调，争取把事关深圳发展的重大项目纳入国家和省“十二五”规划，最大限度地为深圳未来发展争取国家的政策支持。四是严格编制程序，增强民主参与，确保高效率、高水平完成“十二五”规划起草和修改上报工作。

　　（七）全力以赴做好大运会各项筹备工作。今年是大运会筹办的冲刺年，要积极做好各项筹办工作。一是全力推进大运会基础设施建设，确保年底前全面完成66个场馆的新建、维修改造工作，建好大运村及相关配套设施。二是大力推进大运会团队建设和市场运营开发，积极做好各项试运行工作。三是认真贯彻实施城市行动计划细化方案，深入落实大运会的安全保卫、志愿服务、信息通讯、新闻宣传、食品卫生、气象保障等工作。四是积极开展“迎大运、讲文明、树新风”市民素质提升工程，全面普及大运会及体育文化知识，深入开展“百万市民学外语”活动，努力营造全民参与大运、奉献大运、服务大运的氛围。

　　各位代表，深圳新一轮发展的蓝图已经绘就，新30年的伟大征程即将开启，历史赋予我们的使命艰巨而光荣。让我们更加紧密地团结在以胡锦涛同志为总书记的党中央周围，高举中国特色社会主义伟大旗帜，坚持以邓小平理论和“三个代表”重要思想为指导，深入贯彻落实科学发展观，在省委省政府和市委的正确领导下，团结带领全市人民，解放思想，开拓创新，扎实工作，阔步前进，再谱特区建设新篇章，再创深圳发展新辉煌！
